# The Safety of Digital Mental Health Interventions: Systematic Review and Recommendations

**DOI:** 10.2196/47433

**Published:** 2023-10-09

**Authors:** Rayan Taher, Che-Wei Hsu, Chloe Hampshire, Carolina Fialho, Clare Heaysman, Daniel Stahl, Sukhi Shergill, Jenny Yiend

**Affiliations:** 1 Psychosis Studies Department, Institute of Psychiatry, Psychology & Neuroscience (IoPPN), King's College London London United Kingdom; 2 Department of Psychology, University of Bath Bath United Kingdom; 3 London Institute for Healthcare Engineering, King's College London London United Kingdom; 4 Kent and Medway Medical School Canterbury, Kent United Kingdom

**Keywords:** digital, digital therapeutics, mental health, psychological, safety, risk, negative effects, harm, adverse event, risk mitigation, mobile phone

## Abstract

**Background:**

Evidence suggests that digital mental health interventions (DMHIs) for common mental health conditions are effective. However, digital interventions, such as face-to-face therapies, pose risks to patients. A *safe* intervention is considered one in which the measured benefits outweigh the identified and mitigated risks.

**Objective:**

This study aims to review the literature to assess how DMHIs assess safety, what risks are reported, and how they are mitigated in both the research and postmarket phases and building on existing recommendations for assessing, reporting, and mitigating safety in the DMHI and standardizing practice.

**Methods:**

PsycINFO, Embase, and MEDLINE databases were searched for studies that addressed the safety of DMHIs. The inclusion criteria were any study that addressed the safety of a clinical DMHI, even if not as a main outcome, in an adult population, and in English. As the outcome data were mainly qualitative in nature, a meta-analysis was not possible, and qualitative analysis was used to collate the results. Quantitative results were synthesized in the form of tables and percentages. To illustrate the use of a single common safety metric across studies, we calculated odds ratios and CIs, wherever possible.

**Results:**

Overall, 23 studies were included in this review. Although many of the included studies assessed safety by actively collecting adverse event (AE) data, over one-third (8/23, 35%) did not assess or collect any safety data. The methods and frequency of safety data collection varied widely, and very few studies have performed formal statistical analyses. The main treatment-related reported AE was symptom deterioration. The main method used to mitigate risk was exclusion of high-risk groups. A secondary web-based search found that 6 DMHIs were available for users or patients to use (postmarket phase), all of which used indications and contraindications to mitigate risk, although there was no evidence of ongoing safety review.

**Conclusions:**

The findings of this review show the need for a standardized classification of AEs, a standardized method for assessing AEs to statically analyze AE data, and evidence-based practices for mitigating risk in DMHIs, both in the research and postmarket phases. This review produced 7 specific, measurable, and achievable recommendations with the potential to have an immediate impact on the field, which were implemented across ongoing and future research. Improving the quality of DMHI safety data will allow meaningful assessment of the safety of DMHIs and confidence in whether the benefits of a new DMHI outweigh its risks.

**Trial Registration:**

PROSPERO CRD42022333181; https://www.crd.york.ac.uk/prospero/display_record.php?RecordID=333181

## Introduction

Digital mental health interventions (DMHIs) are therapeutic interventions delivered via digital technologies, such as mobile apps, websites, or virtual reality (VR), that aim to improve patients’ mental health [1]. The effectiveness of some of these interventions has been well established and is comparable with that of face-to-face therapies for the treatment of common mental health conditions such as depression and anxiety [1,2]. A meta-analysis of 66 randomized controlled trials (RCTs) found that DMHIs are effective in treating social anxiety and general anxiety disorders [3]. There is also evidence of their effectiveness in reducing paranoia [4].

DMHIs have the potential to improve access to evidence-based mental health therapies and reach individuals who find the traditional mode of delivery for mental health care difficult [3]. Such interventions can help patients overcome the barrier of stigma by giving them the opportunity to privately seek evidence-based care without having to talk to a professional [2]. Digital interventions are medical devices, and as such, their safety is precisely defined within a regulatory context. An intervention is considered safe if its expected benefits (based on quantifiable evidence gathered to date) outweigh any residual risks, once those risks have been mitigated as far as possible [5]. This highlights the importance of systematic identification and measurement of risk before any safety claims can be made. The safety of an intervention is usually captured by the measurement of unwanted occurrences or so-called *negative effects*. The best way to define and categorize these in the context of psychological therapy (as opposed to medical or pharmaceutical) trials has been the subject of discussion in the literature. Some classification schemes have been proposed [6,7], including one specifically for internet interventions [8]; however, there is still no universally accepted rubric. The most widely recognized distinction is between adverse events (AEs) and serious AEs (SAEs). A recent review found that SAEs in psychotherapy trials were fairly consistently conceptualized [7], which may, in part, be due to the strict regulatory and governance requirements around such events. This obliges researchers to use a prescribed definition that is derived from the pharmaceutical industry and relatively universally accepted. For example, the United Kingdom’s Health Research Authority, the United States Food and Drug Administration, and the International Council for Harmonization all consider an SAE to include any event that results in death, disability, incapacity, hospitalization or prolongation of hospitalization, birth defects, or events that might have led to these outcomes were preventative action not taken [9-11]. In contrast, there is considerable heterogeneity in how (nonserious) AEs are conceived, measured, and recorded. In general, AEs include any negative effects or events that occur when a participant is enrolled in a clinical trial. AEs, therefore, encompass a much broader range of less severe possibilities. A systematic review on harm in psychotherapy found that AEs were mentioned significantly more often in pharmacological studies than in psychotherapy studies [7]. In that systematic review, all study protocols that addressed a DMHI (5/115, 4.3%) explicitly considered harm and aimed to assess AEs and SAEs [7]. The review speculated that harm in DMHIs might be more researched compared with face-to-face psychotherapy because of the absence of direct contact with a professional [7].

Previous research on harm concluded that *side effects* are unavoidable in psychotherapy [7,12]. In face-to-face psychotherapy, patients experience approximately 12 AEs per person [7]. The most widely researched risk of mental health interventions is the *deterioration effect* [2]. The deterioration rates reported in DMHIs (12%) are similar to those reported in patients receiving face-to-face therapies [2]. Another risk associated with mental health interventions is novel symptoms—new symptoms not previously experienced by the patient before treatment [13]. Developing dependence on intervention or therapy is a potential risk, which is why therapists usually dedicate sessions toward the end of therapy to prepare patients for termination [13]. Nonresponse (no improvement) is considered a potential negative effect because participation in an ineffective therapy may have prevented access to better alternatives, prolonged the journey to recovery, or hindered recovery [6]. A systematic review found that almost half of patients who receive face-to-face psychotherapy do not experience a significantly positive change in their symptoms posttherapy [7]. AEs, such as deterioration, novel symptoms, and nonresponse are experienced by 5% to 20% of patients [7].

A recent review of study protocols identified problematic heterogeneity in the proposed definitions and assessments of safety, as articulated in the study design stage [7]. Moreover, a recent narrative scoping review explored how AEs are reported in RCTs of DMHIs that are registered in the International Standard Randomized Controlled Trial Number Registry [14]. In this study, we conducted a systematic review of the published safety outcome data to establish the current state of practice, highlight gaps in the literature, and guide future research. Thus, this review aims to answer the following questions.

1. How are the risks and safety of DMHIs currently being assessed? (assessing risk)
2. What are the main reported risks and negative effects of DMHIs? (reporting risk)
3. How do DMHIs mitigate risk in research studies and in the postmarket phase? (mitigating risk)
4. What recommendations can be drawn for future practice based on the current findings?

## Methods

This systematic review followed the PRISMA (Preferred Reporting Items for Systematic Reviews and Meta-Analyses) guidelines [15].

### Eligibility Criteria

#### Inclusion Criteria

We included all study types that addressed the safety, risks, negative effects, or harm of a DMHI, not necessarily as a main outcome, in an adult sample (aged >16 years). This review includes gray literature.

#### Exclusion Criteria

Studies on nonclinical digital interventions that target well-being, such as stress management, were excluded from this review. Studies published in languages other than English and those with research protocols were also excluded.

### Information Sources

A total of 3 scientific databases were searched in June 2022: PsycINFO, Embase, and MEDLINE. The search did not include a limit on the date or language of the publication. A secondary search was then conducted on the web to retrieve any further safety-related information on the interventions identified in this review.

### Search Strategy

The search strategy was run on the 3 selected databases as shown in Textbox 1.

**Search strategy.**

1. appab,ti.
2. apps.ab,ti.
3. digital.ab,ti.
4. wearable device.ab,ti.
5. virtual reality.ab,ti.
6. e-mental health.ab,ti.
7. e-health.ab,ti.
8. internet based.ab,ti.
9. mobile health.ab,ti.
10. telehealth.ab,ti.
11. “risk*”.ab,ti.
12. “safe*”.ab,ti.
13. “negative effect*”.ab,ti.
14. “adverse event*”.ab,ti.
15. harm.ab,ti.
16. mental health.ab,ti.
17. 1 or 2 or 3 or 4 or 5 or 6 or 7 or 8 or 9 or 10
18. 11 or 12 or 13 or 14 or 15
19. 16 and 17 and 18

### Selection Process

One author (RT) conducted the search, removed duplicates, and exported the results to Rayyan, software used to collaborate between reviewers [16]. The retrieved abstracts were independently screened by 4 reviewers (RT, CF, CWH, and C Hampshire) against the inclusion and exclusion criteria. Cohen κ was calculated to assess the interrater reliability between the 4 reviewers and was equal to 0.42, indicating moderate agreement [17]. When the screening was complete, the reviewers met to discuss and resolve any disagreements, which resulted in a unanimous decision in all cases. The full texts of the final agreed list of the included studies were retrieved and screened by RT against the inclusion and exclusion criteria. This was verified by the other reviewers (CF, CWH, and C Hampshire).

### Data Collection Process

One author (RT) extracted relevant data from the studies included in this review.

### Data Items

The following characteristics of each included study were extracted: title; study design; country and year of publication; sample size; group size (if the study included more than one); study aim; and the relevant Participants, Intervention, Comparison, and Outcome (PICO) details.

The following safety-related data reported in the included studies were extracted: the method of assessment used to measure safety, main findings related to the safety of the DMHI, and measures used to minimize risk. Missing data were reported. Publications were also searched to collect any information about AEs that occurred during the study but were not explicitly reported as AEs by the authors.

Publicly available data on the safety of the interventions identified in this review (intended purpose, contraindications, warnings, or any other safety measures) were extracted from a secondary web-based search.

### Study Risk of Bias Assessment

The risk of bias was assessed by 1 reviewer (RT) and verified by another (CWH). Version 2 of the Cochrane risk-of-bias tool for randomized trials (RoB 2) was used to assess the risk of bias in RCTs [18]. RoB 2 estimates the risk of bias that arises because of the randomization process, deviations from intended interventions, missing outcome data, measurement of the outcome, and the selection of the reported result [18]. The Critical Appraisal Skills Programme (CASP) Cohort Study appraisal tool was used to assess the risk of bias in pre-post studies [19]. The tool assesses the risk of bias arising from a study’s validity, bias in recruitment, exposure, outcome measurement, confounding factors, and reporting of results [19].

### Effect Measures

The outcomes of this review were mostly qualitative (information about safety and risks); thus, no effect measure was used in the synthesis of the results.

### Synthesis Methods

A statistical synthesis method such as meta-analysis was not possible because the outcomes were qualitative (information about safety and risks). The results were synthesized and presented in the form of tables. Where appropriate, descriptive statistics such as percentages were used. In addition, we calculated odds ratios and their CIs, for all studies where sufficient data were available, to ascertain whether the probability of experiencing an AE significantly differed between study arms.

## Results

### Study Selection

**Figure 1:**
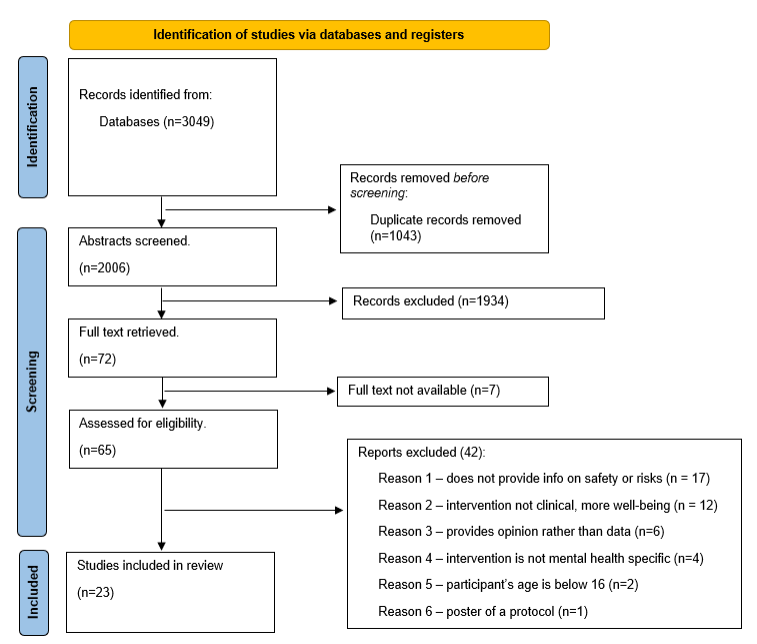
PRISMA (Preferred Reporting Items for Systematic Reviews and Meta-Analyses) flowchart.

The initial search yielded 3049 results of which 23 were included in this review. In all, 1043 duplicates were removed. At the abstract screening stage of 1934, further papers were excluded for the following reasons: do not address digital mental health, do not address mental health, and do not address safety. Of the remaining 72 papers, 65 full-text articles were available and assessed for eligibility. See Figure 1 for the PRISMA flowchart with further details, including reasons for exclusion at this stage.

### Study Characteristics

Of the 23 included studies, 17 (74%) were RCTs and the rest were pre-post studies. The publication dates ranged between 2008 and 2022, with more than 80% (19/23) of the studies published starting from 2018. The sample sizes ranged from 7 to 3755. One-third (8/23, 35%) of the included studies involved collaborations between 2 or more countries. In total, 37 countries were included in the included studies. The most prevalent country was the United Kingdom (10/23, 43%). The countries involved were mainly high income (22/23, 96%) and Western (20/23, 87%). See Table 1 for more details of the study’s characteristics.

Data from the included studies were extracted using PICO criteria:

1. Participants (P): Most (15/23, 65% studies) of the samples in the included studies were clinical: psychosis [4,19-24], depression [25-27], anxiety [28-30], body dysmorphia [31], and eating disorders [32]. Some of the samples represented specific groups, such as first responders who worked during the COVID-19 pandemic [33], veterans with traumatic experiences [34], and physically healthy patients [35,36]. Four of the included studies recruited healthy individuals and screened them for target mental health issues such as suicide [37,38], trauma [39], and insomnia [40].
2. Intervention (I): All interventions in the included studies were digitally delivered. Eleven of them were internet-based programs [25-27,30-35,38,39], 8 were mobile apps [4,19-21,28,29,37,40], 3 were VR-based [22-24], and 1 was social media-based [36].
3. Comparison (C): The 17 RCTs included in this review had different comparison groups. The majority (8/17, 47%) of the comparison groups received treatment as usual [4,20-22,26,34,36,40]. Four comparison groups were waitlist groups [23,29,38,39], 2 were supportive therapy [31,35], 1 was psychoeducation [27], 1 was healthy [28], 1 was a similar neutral intervention [37], and 1 was a similar active intervention [19].
4. Outcome (O): Interventions in the included studies aimed to improve a specific mental health disorder or symptom cluster: depression [25-27,33,35,36], anxiety [22,29,30,33], paranoia [4,23,24], psychosis [19-21,40], suicidal ideation [37,38], posttraumatic stress disorder [33,34,39], eating disorders [32], body dysmorphia [31], and loneliness [28].

Table 1 and Multimedia Appendix 1 [4,20-41] provides more detailed information on the characteristics of the studies and PICO data for each included study.

**Table 1:** Studies’ characteristics.

Study, year	Study design	Total number of participants, N	Study aim	Participants	Intervention	Intervention desired outcome
Arjadi et al [20], 2018	RCT^a^	313	Investigate the efficacy of internet-based behavioral activation with lay counselor support compared with web-based minimal psychoeducation without support for depression in Indonesia.	Those aged 16 y or older or who met the criteria for major depressive disorder or persistent depressive disorder based on the Structured Clinical Interview for DSM-5^b^	Guided Act and Feel Indonesia—the program consists of a series of 8 weekly structured modules that can be completed in 30-45 min per module, including psychoeducation about depression and the basic background of behavioral activation, monitoring mood and behavior or activities, expansion of potential mood-independent pleasurable activities, and building a strategy for relapse prevention	Alleviate depression
Pot-Kolder et al [21], 2018	RCT	116	Investigate the effects of VR-CBT^c^ on paranoid thoughts and social participation	Meets the DSM-4^d^ diagnosis of a psychotic disorder or paranoid ideation in the past month or aged 18-65 y	VR-CBT—consisting of 16 individual therapy sessions	Improve social participation in people with paranoia
Enander et al [22], 2016	RCT	94	Evaluate the efficacy of therapist guided internet based CBT^e^ program for body dysmorphic disorder (BDD-NET) compared with web-based supportive therapy	Aged 18 y or over or a principal diagnosis of body dysmorphic disorder according to the DSM-5	Therapist-guided, internet-based CBT program for body dysmorphic disorder (BDD-NET) - 12 wk long	Decrease the severity of body dysmorphic disorder symptoms
Nissling et al [23], 2020	Single arm (pre- and postdesign)	9	Assess patient experiences; the feasibility, safety, and acceptability; and preliminary effectiveness on anxiety and depression, empowerment, and adherence to treatment in an 8-wk peer-supported iCBT^f^ program for patients with anxiety disorders treated in primary care	Aged 18+ y or meeting the diagnostic criteria for an anxiety disorder	iCBT+peer support−includes 13 different tools, and the treatment consists of 8 modules meant to be completed within 8 wk	Alleviate anxiety
Hamatani et al [24], 2019	Single arm (pre- and postdesign)	7	Evaluate the feasibility of iCBT via videoconference for patients with bulimia nervosa or binge-eating disorder	A primary diagnosis of bulimia nervosa or binge-eating disorder according to the DSM-5 criteria or female or aged 16-65 y	iCBT via videoconference—16 weekly sessions via videoconference with real-time therapist support	Reduce eating disorder symptoms
van Luenen et al, [25], 2018	RCT	188	Investigate the effectiveness of the intervention on depressive symptoms in people living with HIV	HIV positive for at least 6 mo or aged 17+ y or presence of mild to moderate depressive symptoms (PHQ-9^g^ score>4 and <20)	8-wk-long internet-based intervention (available in Dutch and English) consisted of cognitive behavioral therapy, with minimal telephone coaching	Alleviate depression
Freeman et al [26], 2017	RCT	3755	Assess whether treating insomnia leads to a reduction in paranoia and hallucinations	Attending university or positive screen for insomnia, as indicated by a score of 16 or lower on the SCI^h^ or aged 18+ y	Sleepio—digital CBT for insomnia—6 weekly sessions lasting an average of 20 min each	Decrease paranoia and hallucinations
Görges et al [27], 2018	Single arm or pre- and postdesign	81	Investigate the level of satisfaction with a positive psychology web-based training among patients with mild and moderate depression or dysthymia	Aged 18+ y or diagnosis of unipolar depression or dysthymia past or present according to the MIII^i^	“Glück kommt selten allein”—the program comprises 7 modules; each module comprises 2-3 exercises and is meant to be completed within 1 wk	Reduce depression symptoms
Krupnick et al [28], 2017	RCT	34	Assess feasibility, acceptability, and safety of the intervention under study	Aged 18+ y or veteran or had an intake session through the Trauma Services Program	WIRED^j^—10 sessions of a writing intervention	Alleviate PTSD^k^ symptoms
Bragesjö et al [29], 2023	RCT	102	Assess the efficacy of CIPE^l^ as compared with the waiting list in a larger sample and with a longer controlled follow-up period	Adult or exposed to a traumatic event within 2 mo or at least some symptoms of posttraumatic stress	CIPE - 3 wk long	Alleviate PTSD symptoms
Trottier et al [30], 2022	Single arm or pre- and postdesign	21	Assess the feasibility, acceptability, and initial efficacy of RESTORE^m^ in health care workers on the frontline of the COVID-19 pandemic	Canadian health care worker, first responder, or military member who experienced a traumatic or extremely stressful event related to COVID-19 in the course of their work or moderate or more severe symptoms of anxiety, depression, or PTSD symptoms	RESTORE is a web-based guided transdiagnostic intervention, including cognitive-behavioral interventions	Alleviate anxiety, depression, and PTSD
Gumley et al [31], 2022	RCT	74	Establish the feasibility of undertaking a definitive RCT to determine the effectiveness of a blended digital intervention for relapse prevention in schizophrenia	Aged 16+ y or had a schizophrenia or related diagnosis confirmed via case records or experienced a relapse within the previous 2 y	EMPOWER^n^—designed to enable participants to monitor changes in their well-being daily using a mobile phone. Participants could use their own mobile phone.	Prevent relapse
Torok et al [32], 2022	RCT	455	Investigate the efficacy of the life-buoy smartphone app in reducing the severity of suicidal thoughts when compared with an attention-matched smartphone app (LifeBuoy-C)	Between 18 and 25 y of age or in the community (nonclinical sample) or responded in the positive to the question “have you experienced suicidal thoughts in the past 12 months?”	LifeBuoy—a 6-wk self-guided smartphone app based on DBT^o^ to improve emotion regulation and distress tolerance	Decrease the severity of suicidal ideation symptoms
Bucci et al [33], 2018	RCT	36	Assesses the feasibility and acceptability of Actissist, a digital health intervention grounded in the cognitive model of psychosis that targets key early psychosis domains	In current contact with Early Intervention Services or at least 4-wk stabilization of positive symptoms (score <3 on the PANSS^p^ items) or aged 16+ y	Actissist—a 12-wk digital health intervention grounded in the cognitive model of psychosis that targets key early psychosis domains	Alleviate psychotic symptoms
Steare et al [34], 2020	RCT	40	Test the feasibility and acceptability of a RCT to evaluate a smartphone-based self-management tool in Early Intervention in Psychosis services	Aged 16+ y or had experienced at least one episode of psychosis	My journey 3—designed to help Early Intervention in Psychosis service users recognize early warning signs of illness, recognize and monitor symptoms, and create plans for their recovery	Promote self-management posttreatment (psychosis)
Guo et al [35], 2020	RCT	300	Assess the efficacy of a WeChat-based intervention, Run4Love, with a RCT among 300 PLWHD^q^ in China	Aged 18+ y or HIV seropositive or elevated depressive symptoms (measured by the CES-D^r^ ≥16)	Run4Love—the program comprises of 2 major components: the adapted cognitive behavioral stress management course and physical activity promotion	Alleviate depression
Carl et al [36], 2020	RCT	256	Investigate the efficacy of a novel digital CBT program in those with GAD^s^ for outcomes of anxiety, worry, depressive symptoms, sleep difficulty, well-being, and participant‐specific quality of life	Aged 18+ y or a diagnosis of general anxiety disorder	Daylight—digital cognitive behavioral therapy for anxiety	Alleviate anxiety
Lim et al [37], 2019	2 groups (pre-post)	20	Evaluate the acceptability, feasibility, and safety of the program in lonely young people with or without a mental-health diagnosis of social anxiety disorder	Aged 18-25 y or engaged with a current mental health service, general practitioner (or was engaged at time of assessment) or current DSM-5 of social anxiety disorder assessed by the SCID^t^ or UCLA^u^ Loneliness Scale score >38 d	Connect+—the 6-wk program delivers positive psychology content designed to improve relationship quality	Decrease loneliness
Mühlmann et al [38], 2021	RCT	402	Investigate the effectiveness of a guided internet-based self-help program compared with a waiting list control group in reducing sinical ideation	Aged 18+ y with suicidal ideation	Online Living under control self-help program—6 modules of a self-help program for suicidal ideation based on CBT	Decrease suicidal ideation
Yeung et al [39], 2018	RCT	75	Evaluate the acceptability, feasibility, and effectiveness of using the Chinese translated version of MoodGYM (MoodGYM [C]) as an adjunctive intervention for the treatment of depressive symptoms in patients at outpatient clinics in a hospital in Beijing, China	Aged 18+ y or significant depressive symptoms as judged by the patients’ treating clinicians	MoodGYM—4 wk of a web-based computerized CBT	Alleviate depression
Fornells-Ambrojo et al [40], 2008	Single arm or pre- and postdesign	20	Investigate the acceptability and safety of using VR^v^ with individuals with current persecutory delusions	Diagnosis of nonaffective psychosis or a score of at least moderate severity on the Suspiciousness item (P6) of the PANSS or current persecutory delusion	VR—a VR underground train containing neutral characters	Use of VR with individuals with persecutory delusions
Freeman et al [41], 2022	RCT	346	Evaluate the efficacy of an automated VR cognitive therapy (gameChange) to treat avoidance and distress in patients with psychosis, and to analyze how and in whom it might work	Aged 16+ y or with a clinical diagnosis of a schizophrenia spectrum disorder or an affective diagnosis with psychotic symptoms or self-reported difficulties going outside due to anxiety	The gameChange VR therapy (6 wk) aims for participants to relearn safety by testing their fear expectations around other people	Decrease agoraphobia symptoms
Garety et al [4], 2021	RCT	362	Examine the effectiveness of SlowMo therapy in reducing paranoia and in improving reasoning, quality of life and well-being, and to examine its mechanisms of action, moderators of effects and acceptability	Aged 18+ y or persistent (≥3 mo) distressing paranoia or a diagnosis of schizophrenia-spectrum psychosis	SlowMo—consists of 8 individual face-to-face sessions, with each module addressing a specific topic and typically lasting 60-90 min. The therapy was delivered by trained therapists within a 12-wk time frame and was assisted by a web-based application, delivered using the SlowMo web app.	Decrease paranoia

^a^RCT: randomized controlled trial.

^b^DSM-5: Diagnostic and Statistical Manual of Mental Disorders, Fifth Edition.

^c^VR-CBT: virtual reality–based cognitive behavioral therapy.

^d^DSM-4: Diagnostic and Statistical Manual of Mental Disorders, Fourth Edition.

^e^CBT: cognitive behavioral therapy.

^f^iCBT: internet-based cognitive behavioral therapy.

^g^PHQ-9: Patient Health Questionnaire-9.

^h^SCI: sleep condition indicator.

^i^MIII: Mini International Neuropsychiatric Interview.

^j^WIRED: warriors internet Recovery and Education.

^k^PTSD: posttraumatic stress disorder.

^l^CIPE: condensed internet-delivered prolonged exposure.

^m^RESTORE: Recovering from Extreme Stressors Through Online Resources and E-health.

^n^EMPOWER: Early Signs Monitoring to Prevent Relapse in Psychosis and Promote Well-Being, Engagement, and Recovery.

^o^DBT: dialectical behavior therapy.

^p^PANSS: Positive and Negative Syndrome Scale.

^q^PLWHD: people living with HIV and depression.

^r^CES-D: Centre for Epidemiologic Studies-Depression Scale.

^s^GAD: General Anxiety Disorder.

^t^SCID: Structured Clinical Interview for DSM Disorders.

^u^UCLA: University of California, Los Angeles.

^v^VR: virtual reality.

### Risk of Bias in Studies

The RoB 2 tool was used to assess risk of bias in the 17 included RCTs. More than half (9/17, 53%) of the RCTs has some risk of bias. A total of 4 (24%) RCTs had an overall low risk of bias, and another 4 (24%) had an overall high risk of bias. Randomization, missing outcome data, and outcome measurements were the main sources of bias. See Multimedia Appendix 2 [4,18,20-22,25,26,28,29,31-36,38,39,41] for more details on the RoB 2 tool findings. The CASP tool was used to assess the risk of bias in the 6 pre-post studies. The main risk of bias among these studies was identifying and accounting for the confounding variables. See Multimedia Appendix 3 [19,23,24,27,30,37,40] for the results of the CASP tool.

### Results Synthesis

#### Assessing Safety

The main method used to assess safety in the included studies was to collect and report AEs data during clinical trials or other research studies. Most studies (10/23, 57%) actively collected AE data, that is, proactively and systematically asked participants about the occurrence of an AE [4,20,22,24,27,29,31-33,36-38,40]; 2 (9%) studies passively collected AE data, that is, only recorded AE data that were spontaneously reported by participants [26,41]; and more than one-third (8/23, 35%) of the studies did not collect any safety data to assess risk [21,23,25,28,30,34,35,39].

Studies have varied widely in terms of the collection and monitoring of safety data. Some collected only SAE data [33] or only current suicide-related AE data [33,38], whereas others collected self-report measures (no further details were provided) [22,29] or used standardized measures, such as the Fear of Recurrence Scale [31] and the Symptom Checklist [36], while others specifically collected information on symptom deterioration [20]. A study using a VR-based intervention asked participants whether they experienced anxiety, nausea, or disorientation (known side effects of VR) after the intervention [40]. In another study, therapists asked participants about their general physical and mental health and encouraged them to report AEs via email [24]. Lim et al [37] *measured the frequency of AE data* (without specifying how) throughout the study (33 days) and assessed seriousness but found none. Garety et al [4] actively collected AE data over the period of the trial (24-week follow-up) and categorized AEs based on their severity (mild, moderate, severe), relatedness to the intervention, and seriousness. Görges et al [27] actively collected AE data during the 4 main assessment points of the trial and categorized them based on relatedness to the intervention; they also conducted the Patient Health Questionnaire-9 weekly to identify any deterioration in mood or suicidal ideation and conducted the Inventory for the Assessment of Negative Effects of Psychotherapy.

Studies that actively collected risk or safety data varied according to how often they collected the data. Some risk data were collected after every session [24,40], during the main study time points [27,32], weekly [29], mid- and posttreatment [22], postintervention [36], when triggered by a self-report measure [20,38], and throughout the trial plus the follow-up period [4]. Others were unclear about how often they collected the AE data [31,33,37].

#### Reporting Risk

Only one study (EMPOWER [Early Signs Monitoring to Prevent Relapse in Psychosis and Promote Well-Being, Engagement, and Recovery], an app to help prevent relapse in psychosis) reported an SAE related to the DMHI. In this instance, a participant was admitted to the hospital for a physical health complaint that they considered related to feeling overwhelmed by installing the app [31]. Across studies, the main reported AE related to the DMHI was symptom deterioration [27,31,36,38]. Other reported risks include increased anxiety, distress, or depression [22,28,31], triggering traumatic memory [31], increased sleep disturbances [22], and frequent self-monitoring resulting in distress and unhelpful rumination [31]. Technical difficulties were reported in 2 studies [31,36]. In 1 study, 22.6% of the participants felt that they or their problems were not taken seriously by the intervention and 8.1% felt dependent on the program [27].

A total of 7 studies reported that they found no AEs [20,21,24-26,35,37]. However, 2 of these studies reported that participants experienced a deterioration in symptoms [25] and novel symptoms in which sleep treatment led to a sustained increase in mania [26]. Similarly, in another 5 studies, AE data were reported but AEs were not considered by the authors (not reported as such), such as symptom deterioration [23,32,36], changes in medication or treatment [28], and technical problems [34]. See Tables 2 and 3 for more details on the main AE-related findings per study included.

**Table 2:** Studies’ risk-related qualitative findings.

Study, year	AE^a^ or risk or harm or negative effects main findings
Arjadi et al [20], 2018	No AEs were reported in either group.
Pot-Kolder et al [21], 2018	No AEs were reported in either group.
Enander et al [22], 2016	No SAEs^b^ were reported. 15 (32%) participants in the BDD-NET group and 6 (13%) in the supportive therapy group reported mild AEs (increased levels of anxiety and general negative well-being) at the beginning of the trial, which had subsided for everyone at 3 mo, except for 4 participants in the BDD-NET group. Of these, 2 participants reported increased sleep disturbances because of heightened anxiety levels attributed to the exposure exercises, 1 reported depressive mood, and 1 reported that the insight gained throughout the treatment regarding time spent on concerns about appearance was emotionally painful but also enhanced motivation to make changes. After the start of treatment, 1 participant in the BDD-NET group had been prescribed an antidepressant. At follow-up, 3 participants in the BDD-NET group and 2 participants in the control received a new additional treatment. The occurrence of adverse events during treatment was not related to responder status at follow-up (χ^2^_1_=0.9; *P*=.34).
Nissling et al [23], 2020	No SAEs were reported during the treatment period or in the interviews with the participants.
Hamatani et al [24], 2019	No mental or physical AEs were reported.
van Luenen et al [25], 2018	No AEs were reported.
Freeman et al [26], 2017	No AEs were reported.
Görges et al [27], 2018	At follow-up, 14.8% of the participants showed worse depression. Subjective deterioration attributed to the program was at 4.8%. A total of 4 AEs (deteriorations in mood) were *possibly related* to the intervention. According to the INEP^c^, 8.4% of the participants experienced a negative effect at posttreatment, 2.2% reported negative effects due to the program and 6.2% due to other reasons. At follow-up, negative effects rose to 12.2%. However, negative effects attributed to the program decreased to 1.7%. At posttreatment, 22.6% of the participants felt that they or their problems were not taken seriously by the program, and 8.1% of the participants stated that they felt dependent on the content of the program at posttreatment. However, at follow-up, none of the participants felt this way anymore.
Krupnick et al [28], 2017	Two people chose to get in touch with the therapist during the course of the study because of distress following an early session, but others may have simply chosen to stop the intervention in the face of discomfort.
Bragesjö et al [29], 2023	No SAEs were found. In the treatment group, 16 participants (31%) reported a total of 63 AEs (mild to moderate). In the waiting list, 11 participants (21%) reported a total of 35 AEs. Note that in the 27 participants that reported AEs, the average number of reported events were 4 in the treatment group and 3 in the waiting list group. AEs reported: increase in number of intrusive memories of the index event and a previous event, initial symptom exacerbation, increase in depressive symptoms or anxiety, sleep problems, panic attacks, increase in stress, anger or irritability, severe distress during exposure, tiredness, memory impairment, migraine, increase in impulsivity and pain.
Trottier et al [30], 2022	There were no participants withdrawn for safety-related reasons.
Gumley et al [31], 2022	There was a total of 54 AEs, affecting 29 people. Around half of all events across arms were classified as SAEs, and the vast majority of these were anticipated. There was one death during the study. Six events were related to a study procedure, 1 of which was serious (threat made to a member of research). There were 13 app-related AEs, affecting 11 people, 1 of which was serious (brief hospital admission for a physical health complaint, which the service user described as being in part related to feeling overwhelmed by the recent installation of the app—withdrew from the study). Nonserious app-related AEs: 4 instances in which the app caused unhelpful rumination. In 1 of these instances, where the self-monitoring approach was described as counter to the service user’s usual coping strategy of “burying things,” the participant withdrew from the study. Other participants described feeling forced to think about being unwell because of questions in the app, with 1 person suggesting less frequent monitoring in future iterations. Unhelpful rumination of this type was identified by 1 participant as an issue when they were well, whereas a second person was affected when they felt more depressed. Two participants specifically cited increased paranoia because of the app. A further participant identified that personalized question content unhelpfully triggered traumatic memories of psychosis. One participant reported experiencing increased anxiety. In 1 case a participant reported increased worry because of losing their provided mobile phone. One participant experienced distress because of a technical fault. The study also reported on intensity, relatedness, and whether it was anticipated or not.
Torok et al [32], 2022	No SAEs (suicide attempts requiring medical care) were reported.
Bucci et al [33], 2018	No SAEs were reported.
Steare et al [34], 2020	No SAEs were reported.
Guo et al [35], 2020	No AEs were reported.
Carl et al [36], 2020	One participant only, in the treatment group, reported experiencing an AE related to difficulty accessing the intervention, which the participant deemed as distressing and as contributing to increased anxiety. This event was reported to the Ethics Committee. Participants in the treatment group reported significantly fewer occurrences of unwanted symptoms during the study period, including low mood, fatigue or exhaustion, extreme sleepiness, feeling agitated, difficulty remembering things, headache or migraine, difficulty concentrating and focusing on things, reduced motivation or energy, blurred vision, dizziness, and feeling irritable.
Lim et al [37], 2019	No AEs were reported.
Mühlmann et al [38], 2021	In all, 28 (16.8%) participants reported negative effects: 1 unclassifiable, 19 negative emotions or felt worse, 5 increase in suicidal ideation, 3 felt stressed or guilty for not having worked more on the program. Half of the participants that reported negative effects experienced clinically significant improvement in suicidal ideation postintervention. In all, 27 (6.7%) participants—12 in the control group versus 15 in the intervention group—had attempted suicide within the first 6 wk. A total of 44 participants (10.9%) had attempted suicide during the entire period of the study, 22 in each group. Four deaths were reported between postintervention and follow-up, 2 in each group (2 by suicide—1 in the intervention group and 1 in the control group).
Yeung et al [39], 2018	No SAEs were reported.
Fornells-Ambrojo et al [40], 2008	The VR^d^ experience did not raise levels of anxiety or symptoms of simulator sickness. No side effects were reported at the follow-up.
Freeman et al [41], 2022	There were 25 AEs (in 21 patients) in the VR therapy group and 29 AEs (in 19 patients) in the usual care alone group (*P*=.66). There were 12 SAEs (in 9 patients) in the VR therapy group and 8 SAEs (in 7 patients) in the usual care alone group (*P*=.37).
Garety et al [4], 2021	In all, 19 participants in the treatment group and 21 participants in the control group reported 54 AEs (51 serious events, no deaths). More than half of the SAEs were mental health hospital admission or crisis referrals (SlowMo, n=13; TAU^e^, n=16) or physical health crises (SlowMo, n=8; TAU, n=2), none of which was rated as being related to participation in the trial. One SAE in the TAU group was rated as “definitely related” to trial involvement: it involved a complaint made when the research team shared information with the clinical team under a duty of care. None of the AEs were related to the treatment. The types of AEs reported included physical, self-harm, serious violent incident (survivor or accused), referrals to crisis care, admission to psychiatric hospital, and other, along with intensity and relatedness to the intervention.

^a^AE: adverse event.

^b^SAE: serious adverse event.

^c^INEP: Inventory for the Assessment of Negative Effects of Psychotherapy.

^d^VR: virtual reality.

^e^TAU: treatment as usual.

**Table 3:** Studies’ risk-related quantitative findings.

Study, year	Number of participants, N	Intervention group sample size, n (%)	Control group sample size, n	Total number of SAEs^a^, n	Total number of AEs^b^, n	Number of participants who experienced an AE in the intervention group, n (%)	Number of participants who experienced an AE in the control group, n (%)	Values, OR^c^ (95% CI)	*P* value
Arjadi et al [20], 2018	313	159 (50.8)	154 (49.2)	0	0	0 (0)	0 (0)	—^d^	—
Pot-Kolder et al [21], 2018	116	58 (50)	58 (50)	0	0	0 (0)	0 (0)	—	—
Enander et al [22], 2016	94	47 (50)	47 (50)	0	21	15 (31.9)	6 (12.8)	3.2 (1.12-9.19)	.03^e^
Nissling et al [23], 2020	9	N/A^f^	N/A	0	Not reported	N/A	N/A	—	—
Hamatani et al [24], 2019	7	N/A	N/A	0	0	N/A	N/A	—	—
van Luenen et al [25], 2018	188	97 (51.6)	91 (48.4)	0	0	0 (0)	0 (0)	—	—
Freeman et al [26], 2017	3755	1891 (50.4)	1864 (49.6)	0	0	0 (0)	0 (0)	—	—
Görges et al [27], 2018	81	N/A	N/A	0	4	N/A	N/A	—	—
Krupnick et al [28], 2017	34	18 (52.9)	16 (47.1)	0	Not reported	N/A	N/A	—	—
Bragesjö et al [29], 2023	102	51 (50)	51 (50)	0	98	16 (31.4)	11 (21.6)	1.66 (0.68-4.05)	.26
Trottier et al [30], 2022	21	N/A	N/A	0	Not reported	N/A	N/A	—	—
Gumley et al [31], 2022	74	42 (56.8)	31 (41.9)	27	54	29 (69)	25 (80.6)	0.54 (0.18-1.62)	.27
Torok et al [32], 2022	455	228 (50.1)	227 (49.9)	0	Not reported	N/A	N/A	—	—
Bucci et al [33], 2018	36	24 (66.7)	12 (33.3)	0	Not collected	N/A	N/A	—	—
Steare et al [34], 2020	40	20 (50)	20 (50)	0	Not reported	N/A	N/A	—	—
Guo et al [35], 2020	300	150 (50)	150 (50)	0	0	0 (0)	0 (0)	—	—
Carl et al [36], 2020	256	128 (50)	128 (50)	0	1	1 (0)	0 (0)	3.02 (0.12-74.92)	.45
Lim et al [37], 2019	20	9 (45)	11 (55)	0	0	0 (0)	0 (0)	—	—
Mühlmann et al [38], 2021	402	196 (48.8)	206 (51.2)	44	72	22 (11.2; SAE only)	22 (10.7; SAE only)	—	—
Yeung et al [39], 2018	75	37 (49.3)	38 (50.7)	0	Not reported	N/A	N/A	—	—
Fornells-Ambrojo et al [40], 2008	20	N/A	N/A	0	0	N/A	N/A	—	—
Freeman et al [41], 2022	346	174 (50.3)	172 (49.7)	20	54	30 (17.2)	26 (15.1)	1.17 (0.66-2.08)	.59
Garety et al [4], 2021	362	181 (50)	181 (50)	51	54	25 (13.8)	26 (14.4)	0.96 (0.53-1.73)	.88

^a^SAE: serious adverse event.

^b^AE: adverse event.

^c^OR: odds ratio.

^d^Not available.

^e^Significant at *P*<.05.

^f^N/A: not applicable.

One study analyzed whether negative effects (feeling worse, increased suicidal ideation, feeling stressed, or guilty for using the program) affected the effectiveness of the intervention. They found that out of the 14% (n=28) of people who reported a negative effect, 50% (n=14) experienced clinically significant improvement [38]. However, without a suitable comparator condition (eg, the proportion experiencing improvement in the absence of negative effects), this finding is difficult to interpret.

#### Odds Ratio Secondary Data Analysis

The data (number of participants who experienced an AE per group) in Table 2 shows that 6 (35%) out of the 17 RCTs collected and reported sufficient data to calculate odds ratios and CIs. Two of these studies conducted similar analyses by statistically comparing the occurrence of AEs between the treatment and control groups, and both found no significant differences [22,41], consistent with our odds ratio findings. In the remaining 4 studies, our odds ratio analysis revealed that one study showed a significantly elevated risk of harm. In this case, those receiving the intervention were over 3 times more likely to experience an AE during the study, compared with those in the control arm. In contrast, the authors reported that the number of AEs experienced during the study was not related to the responder status at follow-up (*P*=.34) [22]. The authors concluded that their DMHI was safe, with no occurrence of SAEs.

#### Mitigating Risk—in the Research Stage

Researchers and authors differ in the actions taken to minimize risk in their studies. One study explicitly described providing participants with support resources [20]. One study excluded participants who could not provide contact details for an emergency contact person [38]. Most of the studies minimized risk by excluding specific clinical groups from their samples such as individuals with a high risk of suicide [20,22-24,28-30,32,37-39,41], individuals with bipolar or manic disorder [20,22,24,29,32,36,39], individuals experiencing psychosis or diagnosed with a psychotic disorder [20,22,24,28,29,32,36,37,39], individuals with a personality disorder [4,22,37,41], those at high risk of self-harm [37,39], and individuals with severe depression [29]. Overall, 9 (39%) of the 23 included studies did not explicitly report any other safety precautions [21,25-27,31,33-35,40].

#### Mitigating Risk—Postmarket

A web-based secondary search was conducted to identify any further available safety information for the interventions under review. Six (26%) of the 23 interventions in this review were accessible to users or patients [22,26,30,33,36,39]. The search identified the safety information for all 6 interventions. This information was available on app stores [33], interventions’ websites [30,39], in the instructions for use [26,36], privacy policy [26], and a publicly available analysis that was performed by the Improving Access to Psychological Therapies program in the United Kingdom for one of the products [22].

Different safety strategies have been implemented for different interventions. All 6 interventions identified the indications and contraindications for use. Only 2 studies specified the minimum age required for users to use the intervention [26,39]. One intervention (BDD-NET, an internet-based cognitive behavioral therapy program for body dysmorphic disorder) highlighted the need for therapists to be trained before administering the program [22]. BDD-NET is also the only intervention that sends trigger alerts to therapists regarding potential risks [22]. The MoodGYM program suggested that its users contacted a health professional if they scored above 2 to 3 on their depression quiz [39]. Actissist (an intervention for psychosis) specified that its users should only use the intervention under the supervision of a qualified health care professional [33]. Some interventions highlighted that they are not substitutes for therapy or medication [26,30,33,36]. Three interventions stated that they were not intended for emergency use and provided their users with resources in the case of an emergency [26,36,39]. These interventions also encouraged users to consult their physician if their symptoms worsened [26,36,39]. Finally, only 2 interventions made their AE data publicly available to users through their instructions for use (not in an academic publication) [26,36]. There was no information available on whether any of these 6 interventions continued to monitor risk and update their safety procedures postmarket phase.

## Discussion

This is the first review to systematically evaluate literature on the safety of DMHIs. It aimed to better understand how DMHIs assess, report, and mitigate risk and identify any emerging recommendations, building on previous work [6,7].

### Principal Findings

#### Assessing Risk

Only two-thirds (15/23, 65%) of the studies included in this review assessed risk, although this is an improvement compared with the findings of a systematic review conducted in 2017, where none of the 9 included studies assessed safety or the occurrence of AEs [42,43]. We recommend that safety assessment should be systematically assessed in every DMHI study (recommendation 1). However, this is not a new recommendation. In 2014, colleagues were urging researchers to “...systematically probe for negative effects whenever conducting clinical trials involving Internet interventions, as well as to share their findings in scientific journals” [7]. Studies varied in how often they collected risk data, whether it was weekly, after every session, until follow-up, or just postintervention. The minimal approach was the passive collection of spontaneously reported AEs. By contrast, the most proactive actively collected data on any AE (irrespective of relatedness to the intervention) using a regularly repeated measure or set of questions. To effectively assess safety, research using DMHIs should actively and regularly collect safety data both throughout intervention delivery and after the intervention until follow-up (recommendation 2). Others have suggested minimum midtreatment, at the end of treatment, and at one follow-up time point [8]. The frequency of safety data collection should be as high as possible while balancing the burden on the research team and participants. In the digital context, there is potential for automating negative effect reporting within the technology itself [14]. The collection of sufficient safety data observations is also a prerequisite for conducting formal statistical analyses of these data, a point we return to below.

The methods used to assess safety varied widely between studies and included standardized instruments, bespoke questionnaires, or unspecified “self-report.” This review highlights the need for a minimum agreed upon standard for assessing risk data in DMHIs. A recent narrative scoping review on identifying and categorizing AEs in DMHIs suggested using the digital functionality of DMHIs to streamline the process of detecting harm and AEs in these interventions [44]. Regardless, the methods and instruments used to measure safety should be reported in sufficient detail to permit replication (recommendation 3).

In the analyses of collected risk data, only 2 studies conducted statistical analyses to compare whether the occurrence of AEs significantly varied between the control and treatment groups. Neither found statistical support for significant differences. This provides evidence consistent with the treatments being safe [22,41]. The odds ratio analysis presented in this review illustrates another possible comparison that could add value but has not yet been used. For example, according to this metric, participants in the intervention group of one study were 3 times more likely to experience AEs compared with those in the control group; however, unaware of this, the study authors deemed the DMHI safe [22]. Thus, rates of harm should be statistically compared between study arms and across different studies using standardized quantitative metrics (recommendation 4). Irrespective of the specific analytical approach used, formal statistical analysis of AE data, interpreted in the wider context of a study, is a key requirement to allow stakeholders to make fully informed, evidence-based judgments about safety. Our suggestions include reporting the mean (and median) SAEs per participant for each study, listing the number of SAEs for each participant, and reporting odds ratios, as illustrated here.

#### Reporting Risk

Symptom deterioration was one of the main risks reported in this review, similar to face-to-face therapies [13,45]. Some studies have argued that short-term and transient deterioration of symptoms during psychological therapy can be a normal and integral part of treatment [46,47]. Others argue that AEs are negatively correlated with positive therapy outcomes and are not expected to be a lasting consequence of effective therapy [48,49]. If deterioration is an expected part of the treatment, this should be acknowledged a priori, and a quantitative threshold is identified to define this as an AE (recommendation 5). The Australian National Safety and Quality Digital Mental-Health Standards state “Recognizing and responding to acute deterioration” as one of their standards [50]. Moreover, a review on the identification of AEs in DMHIs suggested the development of a digitally delivered symptom checklist and setting predefined cutoffs to detect symptom deterioration in DMHIs [44].

This review also highlighted the lack of agreement and conceptual clarity regarding which events are considered AEs. All 7 studies in this review that reported “no AEs” in their publication reported events that indicated an AE, such as deterioration in symptoms and novel symptoms, but did not categorize them as AEs. This finding is in line with a recent review on the topic, which found that authors presented events that indicated an AE or SAE but did not categorize it as such [44]. The authors of that review speculated that the difficulties faced in classifying AEs in DMHIs were due to the lack of guidelines [44]. Researchers have previously highlighted the need to improve the classification of AEs in DMHIs [7,14]. A consensus classification framework for AEs would allow the field to more clearly establish whether the benefits of a new DMHI outweigh the risks, and how it compares to alternative treatment options.

#### Mitigating Risk

The main method used to mitigate risk during DMHI trials and research studies was the exclusion of at-risk groups, such as those at high risk of suicide or those diagnosed with a specific mental health condition or severity of the condition. The literature shows that individuals with suicidal thoughts and behaviors are routinely excluded from mental health research because of concerns about safety or lack of resources to implement effective risk management measures [1].

Exclusive practices are unintentionally reinforced by regulatory requirements that prioritize safety over inclusivity, resulting in narrow and specific indications for use. This is justifiable for new, untested treatments. However, once basic safety parameters are established, the ongoing exclusion of more vulnerable groups is a serious limitation that the research community must address. Exclusive practices do not reflect the real world, limit generalizability, and deny the possible benefits of DMHIs for more *risky* segments of the target population [1]. The research community is urged to seek future opportunities to assess the safety of their interventions in specific groups that they have previously excluded [51]. Safety and efficacy should be assessed in high-risk groups with appropriate safeguards (recommendation 6).

The included studies mainly used their risk-related findings to conclude the safety of the DMHI under study rather than to inform future practice, product iterations, or safety measures. Only one study used risk data to make suggestions for possible safety measures needed in similar DMHIs in the future. The authors suspected that one of the modules that required engaging with people was responsible for the deterioration experienced by their users [27]. As a result, they suggested excluding similar exercises from similar interventions or introducing them toward the end to reduce possible negative effects [27]. In addition, most of the safety information provided publicly to users, although necessary, was not provided by the risk data collected in their respective studies. For example, although research studies excluded at-risk groups when assessing safety, these groups were often not excluded when the DMHI was implemented. Safety data collected during the research phase should be used to inform risk-mitigation postmarket (recommendation 7).

It seems that *assessing risk* in the research phase is an independent process from *mitigating risk* in the postmarket phase. We speculate that this was due to the quality of the safety data collected during the research phase. To use risk data from the research phase of the DMHI in the mitigation process, studies need to enhance the assessment and analysis of safety in the ways recommended above. In addition, research is needed to assess the effectiveness of common methods used to mitigate risks in psychotherapy in a digital context. Ideally, such findings can result in a list of actions to mitigate specific identified risks that have been proven to be effective.

### Limitations

This study has some limitations. Most RCTs included in this review had some concerns about the risk of bias. More than one-quarter (6/23, 26%) of the included studies were not RCTs, and thus had concerns around “confounding variables.” Finally, most of the included studies were conducted in the high-income Western world; thus, their generalizability is limited.

Only studies published in English were included in this review. Only studies including adult populations were included in this review. In addition, our search results were dictated by the words included in our search strategy (safety, risk, negative effects, AEs, etc), meaning that we only extracted and reviewed studies that assessed risk. This may have led to an inflation of studies that assessed and reported AEs, as studies that did not explicitly state that they assessed the risks of a DMHI (eg, did not label their findings as such) will have been excluded.

### Conclusions

This review highlights that the approach to assessing and mitigating risk in DMHIs varies widely and is sometimes inadequate. To formulate a set of 7 recommendations (see Textbox 2 for the full list), we focused on relatively indisputable points that are specific, measurable, and achievable. This review also endorses the widely recognized need for collaboration between key stakeholders, including academics, health professionals, developers, product managers, commissioners, and regulatory bodies, to reach a consensus on how the risks of DMHI should be assessed, reported, and mitigated. Standardized definitions and guidelines are needed to provide professionals with tools to reliably assess the safety of their interventions, manage risk, and protect patients and users from unnecessary harm. Finally, research on patients or users’ experiences and their concerns about the safety of DMHIs is needed, as it is currently nonexistent.

**Full list of recommendations.**

- Recommendation 1: Safety assessment as standard—risk and safety should be systematically and proactively assessed in every digital mental health intervention (DMHI) study
- Recommendation 2: Frequency of safety assessment—risk and safety assessments should take place at prespecified, regular intervals throughout both the intervention and follow-up phases
- Recommendation 3: Measures of safety assessment—the methods and instruments used to measure safety should be reported in sufficient detail to permit replication
- Recommendation 4: Statistical comparison—rates of harm should be statistically compared both between study arms and across different studies using standardized quantitative metrics
- Recommendation 5: Symptom Deterioration—if deterioration is an expected part of treatment, this should be acknowledged a priori, and a quantitative threshold identified for defining this as an adverse event
- Recommendation 6: Inclusivity—safety and efficacy should be assessed in high-risk groups, with appropriate safeguards
- Recommendation 7: Postmarket mitigation—safety data collected during the research phase should be used to inform risk mitigation postmarket

## Appendix

Multimedia Appendix 1 Other studies details.

Multimedia Appendix 2 Version 2 of the Cochrane risk-of-bias tool for randomized trials (RoB 2) generated an output and legend.

Multimedia Appendix 3 Critical Appraisal Skills Programme tool results.

Multimedia Appendix 4 PRISMA Checklist.

## Abbreviations

AE: adverse event
CASP: Critical Appraisal Skills Programme
DMHI: digital mental health intervention
EMPOWER: Early Signs Monitoring to Prevent Relapse in Psychosis and Promote Well-Being, Engagement, and Recovery
PICO: Participants, Intervention, Comparison, and Outcome
PRISMA: Preferred Reporting Items for Systematic Reviews and Meta-Analyses
RCT: randomized controlled trial
RoB 2: Version 2 of the Cochrane risk-of-bias tool for randomized trials
SAE: serious adverse event
VR: virtual reality
