# Supplementary material for: The Safety of Digital Mental Health Interventions: Systematic Review and Recommendations
Source: JMIR Ment Health. 2023 Oct 9;10:e47433. doi: 10.2196/47433 (PMC10594135; doi:10.2196/47433)
Supplement: Multimedia Appendix 1 [file mental_v10i1e47433_app1.docx]

**Appendix 1.** Other studies’ details

| **Study, year** | **Countries** | **Intervention group Sample size** | **Control group sample Size** | **Comparison group (C)** |
| --- | --- | --- | --- | --- |
| Arjadi et al [20], 2018 | Indonesia, India, Netherlands, USA | 159 | 154 | Online psychoeducation group |
| Pot-Kolder et al [21], 2018 | Netherlands | 58 | 58 | Waiting list |
| Enander et al [22], 2016) | Sweden, UK | 47 | 47 | Online supportive therapy |
| Nissling et al [23], 2020) | Sweden | N/A | N/A | None |
| Hamatani et al [24], 2019 | Japan | N/A | N/A | None |
| van Luenen et al [25], 2018 | Netherlands | 97 | 91 | Weekly attention only from a coach |
| Freeman et al. [26], 2017) | UK | 1891 | 1864 | Treatment as usual |
| Görges et al. [27], 2018 | Germany | N/A | N/A | None |
| Krupnick et al. [28], 2017 | US | 18 | 16 | Treatment as usual |
| Bragesjö et al. [29], 2021 | Sweden | 51 | 51 | Waiting list |
| Trottier et al. [30], 2022 | UK | N/A | N/A | None |
| Gumley et al. [31], 2022 | UK, Australia, Canada | 42 | 31 | Treatment as usual |
| Torok et al. [32], 2022 | UK, Australia, Canada | 228 | 227 | LifeBuoy-C- general (nontherapeutic) information on a range of health and lifestyle topics. |
| Bucci et al. [33], 2018 | Australia | 24 | 12 | ClinTouch - symptom-monitoring control |
| Steare et al. [34], 2020 | UK | 20 | 20 | Treatment as usual |
| Guo et al. [35], 2020 | UK | 150 | 150 | Treatment as usual |
| Carl et al. [36], 2020 | China, USA | 128 | 128 | Waitlist control |
| Lim et al. [37], 2019 | Australia, USA | 9 | 11 | Participants with no mental health conditions |
| Mühlmann et al. [38], 2021 | Denmark | 196 | 206 | Waitlist control |
| Yeung et al. [39], 2018 | USA, China, Australia | 37 | 38 | Treatment as usual |
| Fornells-Ambrojo et al. [40], 2008 | UK, Spain | N/A | N/A | None |
| Freeman et al. [41], 2022 | UK | 174 | 172 | Treatment as usual |
| Garety et al. [4], 2021 | UK | 181 | 181 | Treatment as usual |
