# Supplementary material for: The Safety of Digital Mental Health Interventions: Systematic Review and Recommendations
Source: JMIR Ment Health. 2023 Oct 9;10:e47433. doi: 10.2196/47433 (PMC10594135; doi:10.2196/47433)
Supplement: Multimedia Appendix 2 [file mental_v10i1e47433_app2.docx]

**Appendix 2.** Version 2 of the Cochrane risk-of-bias tool for randomized trials (Rob 2) generated output plus legend [18]

| **Study ID** | **Domain 1** | **Domain 2** | **Domain 3** | **Domain 4** | **Domain 5** | **Overall risk of bias** |  | 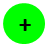Low risk |
| --- | --- | --- | --- | --- | --- | --- | --- | --- |
| Arjadi et al [20], 2018 |  |  |  |  |  |  |  | Some concerns |
| Pot-Kolder et al [21], 2018 |  |  |  |  |  |  |  | High risk |
| Enander et al [22], 2016 |  |  |  |  |  |  |  |  |
| van Luenen et al. [25], 2018 |  |  |  |  |  |  |  |  |
| Freeman et al [26], 2017 |  |  |  |  |  |  |  |  |
| Krupnick et al [28], 2017 | 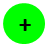 | 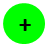 |  |  |  | 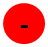 |  |  |
| Bragesjö et al [29], 2021 |  |  |  |  |  |  |  |  |
| Gumley et al [31], 2022 |  |  |  |  |  |  |  |  |
| Torok et al [32], 2022 |  |  |  |  |  |  |  |  |
| Bucci et al [33], 2018 |  |  |  |  |  |  |  |  |
| Steare et al. [34], 2019 |  |  |  |  |  |  |  |  |
| Guo et al [35], 2020 |  |  |  |  |  |  |  |  |
| Carl et al [36], 2020 |  |  |  |  |  |  |  |  |
| Mühlmann et al [38], 2021 |  |  |  |  |  |  |  |  |
| Yeung et al [39], 2018 |  |  |  |  |  |  |  |  |
| Freeman et al [41], 2022 |  |  |  |  |  |  |  |  |
| Garety et al, [4], 2021 |  |  |  |  |  |  |  |  |

Note: Domain 1: Bias arising from the randomization process. Domain 2: Bias due to deviations from the intended intervention. Domain 3: Bias due to missing outcome data. Domain4: Bias in measurement of outcome. Domain 5: Bias in selection of the reported result. The legend to the right of the figure shows what level of bias each circle corresponds to. Further information on how the tool works can be found here <https://methods.cochrane.org/risk-bias-2>.
