# Supplementary material for: The Safety of Digital Mental Health Interventions: Systematic Review and Recommendations
Source: JMIR Ment Health. 2023 Oct 9;10:e47433. doi: 10.2196/47433 (PMC10594135; doi:10.2196/47433)
Supplement: Multimedia Appendix 3 [file mental_v10i1e47433_app3.docx]

**Appendix 3.** Critical Appraisal Skills Programme (CASP) Tool Results [19]

| **CASP TOOL** | Nissling et al [23], 2020 | Hamatani et al [24], 2019 | Görges et al [27], 2018 | Trottier et al [30], 2022 | Lim et al [37], 2019 | Fornells-Ambrojo et al [40], 2008 |
| --- | --- | --- | --- | --- | --- | --- |
| **1.** Did the study address a clearly focused issue? | Yes | Yes | Yes | Yes | Yes | Yes |
| **2.** Did the authors use an appropriate method to answer the question? | No | Yes | Yes | Yes | Yes | Yes |
| **3.** Was the cohort recruited in an acceptable way? | Yes | Yes | Yes | Yes | No | Yes |
| **4.** Was the exposure accurately measured to minimize bias? | Yes | Yes | Yes | Yes | Yes | Yes |
| **5.** Was the outcome accurately measured to minimize bias? | No | Yes | Yes | Yes | No | No |
| **6a.** Have the authors identified all important confounding factors? | No | No | Yes | No | No | No |
| **6b.** Have they taken account of the confounding factors in the design and/or analysis? | No | No | No | No | No | No |
| **7a.** Was the follow up of subjects complete enough? | Yes | Yes | Yes | No | Yes | No |
| **7b.** Was the follow up of subjects long enough? | Yes | Yes | Yes | No | Yes | No |
| **8.** What are the results? | Peer support in digital treatment seems to be a safe and acceptable intervention. | ICBT via videoconference is feasible in Japanese patients with bulimia nervosa and binge-eating disorder | Overall satisfaction was promising. | RESTORE was found to be feasible and safe, and led to statistically significant and large effect size improvements in anxiety, depression, and PTSD symptoms. | Those with social anxiety disorder reported less acceptable ratings on outcomes. Those with social anxiety disorder yielded more attrition. There were no safety. | The study indicates that brief experiences in VR are safe and acceptable to people with psychosis. |
| **9.** How precise are the results? | Small sample and qualitative data. | Precise. | Precise. | The results on safety and effectiveness require a larger study. | Precise, except safety (not enough detail provided). | Precise. |
| **10.** Do you believe the results | Yes | Yes | Yes | Yes | Yes | Yes |
| **11.** Can the results be applied to the local population? | No | No | Yes | Yes | No | Yes |
| **12.** Do the results of this study fit other available evidence? | Yes | Yes | No | Yes | Can't Tell | Yes |
